# Targeted therapies in neoadjuvant treatment for gastroesophageal cancer

**DOI:** 10.3332/ecancer.2025.1921

**Published:** 2025-06-03

**Authors:** Berenice Freile, Andrés Rodriguez, Greta Catani, Marcos Bortz, Luisina Bruno, Juan M O’Connor, Federico Esteso

**Affiliations:** Gastrointestinal Department, Clinical Oncology, Instituto Alexander Fleming, CABA 1426, Argentina

**Keywords:** neoadjuvant, gastric cancer, esophageal cancer, immunotherapy, target therapy

## Abstract

Gastroesophageal cancers are among the most prevalent cancers globally and represent the third leading cause of cancer-related mortality worldwide. Surgical resection remains the primary curative approach for localised and locally advanced stages, but its effectiveness is limited for locally advanced diseases, evidenced by a low 5-year survival rate of around 25%. High relapse rates post-surgery, particularly in Western populations, necessitate the use of neoadjuvant, adjuvant or perioperative strategies involving chemotherapy and radiation to improve surgical outcomes. Neoadjuvant chemoradiation therapy has demonstrated a significant improvement in overall survival. Recent advances have identified several target genes and pathways involved in the pathogenesis and progression of these cancers, leading to the development of targeted drugs, including immunotherapy, anti-HER-2 antibodies and anti-vascular endothelial growth factor receptor antibodies. These targeted therapies are emerging as promising interventions for better patient outcomes and personalised treatment approaches and, therefore, could eventually evolve into a novel therapeutic regimen for gastroesophageal cancer.

## Introduction

Gastroesophageal cancers, encompassing malignancies of the esophagus, gastroesophageal junction (GEJ) and stomach, are among the most prevalent cancers globally. Their incidence shows geographical variation, with rates spanning from 3.0 to 32.2 per 100,000 individuals, influenced by factors such as country and gender. Collectively, they stand as the third leading cause of cancer-related mortality worldwide [1].

Localised and locally advanced disease accounts for 18% of esophageal cancers (EC) [2] and 28% of gastric cancers (GC) [3] at the time of diagnosis. Primary tumour location and histology determines management in this setting [4].

While surgical resection remains the primary curative approach, its efficacy is limited in the context of locally advanced disease, with a mere 25% 5-year survival rate [5] and over 30% of patients in the Asian population and up to 70% in the Western population relapse even after complete resection and adjuvant therapies [6, 7]. Therefore, neoadjuvant, adjuvant and perioperative strategies incorporating chemotherapy (CT) and radiation are crucial to optimise surgical outcomes. Neoadjuvant chemoradiation therapy has demonstrated significant improvements, with a 10-year overall survival (OS) rate reaching 38% [8], while perioperative CT achieves a notable 5-year OS rate of 48.5% [9]. Neoadjuvant therapy not only provides proven benefits but is also established as the standard of care, facilitating tumour burden reduction, preoperative tumour response assessment and ultimately enhancing clinical outcomes.

Several target genes and pathways implicated in the pathogenesis and progression of GC and EC have been identified, driving rapid developments in therapeutic drug exploration. These targeted drugs primarily encompass immunotherapy (IO), anti-human epidermal growth factor receptor-2 (HER-2) antibodies and anti-vascular endothelial growth factor receptor (VEGFR) antibodies. Emerging as promising approaches for improved patient outcomes and tailored interventions [10].

## Neoadjuvant anti-VEGF

Tumour angiogenesis plays a crucial role in cancer cell proliferation and metastasis. In GC, numerous clinical trials investigating anti-angiogenic therapies. In the open-label phase II/III trial ST03, a total of 1,063 patients diagnosed with resectable gastric, GEJ or EC were included. These patients were randomly assigned to receive either perioperative CT (epirubicin, cisplatin and capecitabine) alone or in combination with bevacizumab. The results indicated that the addition of bevacizumab to perioperative CT failed to enhance rates of R0 resection or 3-year survival compared to CT alone (61% versus 64%, *p* = 0.47; 48.1% versus 50.3%, *p* = 0.36, respectively). Furthermore, the administration of bevacizumab was associated with a higher incidence of compromised wound healing [11].

However, a different approach yielded more promising results. Zheng et al [12] reported the findings of a single-arm phase II clinical trial showcasing encouraging results with the preoperative utilisation of the SOX (S-1 and oxaliplatin) alongside apatinib for locally advanced gastric adenocarcinoma (AC). Among a total of 29 enrolled patients, the objective response rate achieved was 79.3% (95% CI, 60.3%–92.0%) and the disease control rate was 96.6% (95% CI, 82.2%–99.9%). The pathologic complete response rate was 13.8% (95%CI, 1.2%–26.3%). Notably, the documented adverse reactions were deemed manageable and well-tolerated [12].

Additionally, Lin et al [13] explored the same combination therapy in a multicenter, prospective, single-group, open-label, phase II study. In this trial, 48 eligible patients received perioperative treatment with apatinib plus SOX, resulting in a pathological response rate of 54.2% (95% CI, 39.2%–68.6%). Interestingly, tumours located in the upper one-third of the stomach exhibited a better response, suggesting potential site-specific effects [13].

Ramucirumab, an additional anti-VEGFR agent, underwent evaluation in this scenario. The RAMSES/5-fluorouracil, oxaliplatin, leucovorin and docetaxel (FLOT)7 trial, a randomised Phase II/III investigation, explored the incorporation of the VEGFR-2 inhibitor ramucirumab into FLOT as perioperative therapy for resectable esophageal gastric adenocarcinoma (EGA). As no discernible difference in the pathological complete response (pCR)/pathological subtotal response rate between the treatment arms was found, the trial did not advance to phase III. Nevertheless, the combination arm exhibited a significantly higher R0 resection rate compared to FLOT alone (82% versus 96%; *p* = 0.009). Furthermore, a trend towards improved median disease-free survival (DFS) by 9 months was observed (HR 0.75, *p* = 0.218) [14].

## Neoadjuvant anti HER2 therapy

The overexpression and amplification of HER2 is detected in approximately 15%–20% of EGA cases, particularly in tumours originating from the GEJ with an intestinal tumour type according to Lauren classification. In the advanced setting, HER2 positivity serves as a reliable predictive marker for treatment with trastuzumab (T) when combined with platinum-based CT and pembrolizumab in patients with combined positive score (CPS) greater or equal to one [15], resulting in a survival benefit for patients. However, currently, no HER2-directed therapy is available in the neoadjuvant or perioperative setting for gastric, GEJ or EC [16].

In the NRG Oncology/RTOG-1010 phase III trial, 203 untreated HER2-overexpressing esophageal AC patients from the USA were enrolled. They were randomly assigned to receive CT (paclitaxel plus carboplatin) and radiotherapy (RT) ± T as perioperative treatment. The experimental arm did not significantly improve DFS (HR = 0.99, 95% CI = 0.71–1.39, *p* = 0.97) or OS (HR = 1.04, 95% CI = 0.71–1.50, *p* = 0.85) [17].

A phase II, one-arm trial, presented by Hofheinz et al [18, 19] investigated the combination of T with CT. In this instance with 5-fluorouracil, oxaliplatin, leucovorin and docetaxel (FLOT) as perioperative treatment in patients with locally advanced EGA. Among the 56 enrolled patients in this trial, PRETARCA, the R0 resection rate was 92.9%. pCR was observed in 12 patients (21.4%). The median DFS was 42.5 months and the 3-year OS rate was 82.1% [18]. After this trial, a randomised phase II/III trial with the incorporation of pertuzumab (P) to T and FLOT was designed by the same authors. This trial was closed prematurely, without transition into phase III, after the results of the JACOB trial were reported. Eighty-one patients were randomly assigned to perioperative FLOT alone or combined with T and P during the phase II part. The pCR rate was significantly improved in the experimental arm (A: 12% versus B: 35%; *p* = 0.02). Likewise, the rate of pathologic lymph node negativity was higher with T + P (A: 39% versus B: 68%) [19].

Another trial that explored the efficacy of combining T alone or with P with perioperative CT for gastric and GEJ cancer, was the phase II EORTC 1203 INNOVATION trial. Conducted collaboratively by the Korean Cancer Study Group and the Dutch Upper GI Cancer group, although this trial was prematurely terminated due to slow accrual, 172 patients were randomised in a 1:2:2 ratio to receive CT alone, CT + T or CT + T + P. Combination with CT + T + P showed lower compliance than the other arms (only 81.3% completed neoadjuvant treatment versus 90.9% and 92.2%), predominantly owing to toxicity. Even though the primary endpoint analysis did not meet the pre-specified criteria of efficacy for the combination of CT + T + P, the addition of P to T and CT resulted in an increased major pathological response rate (MpRR) of 13.7% (80% CI: (0.7%, 26.7%), one-sided *p* = 0.099) [20].

The EPOC2003 trial, assessed trastuzumab-deruxtecan (T-DXd), an antibody-drug conjugate containing a humanised anti-HER2 IgG1 monoclonal antibody with the same amino acid sequence as trastuzumab, covalently linked to a topoisomerase I inhibitor. This phase II study included 27 Japanese patients with locally advanced HER2-positive gastric and GEJ AC. Treatment consisted of three cycles of T-DXd administered every 3 weeks, followed by surgery. Twenty-six patients completed the three planned courses of T-DXd, while one discontinued due to toxicity. R0 resection was achieved in 25 patients. The MpRR was a modest 14.8% [21].

## Neoadjuvant IO

While not precisely a targeted therapy, immune checkpoint inhibitors (ICIs), primarily targeting programmed cell death protein 1 (PD-1) or programmed cell death ligand 1 (PD-L1), aim to reinvigorate CD8+ cytotoxic T cells to identify and eliminate (neo)antigens presented by tumour cells or antigen-presenting cells. Immune checkpoints are membrane proteins that regulate immune responses physiologically. Tumour cells subvert immune surveillance by impairing neoantigen presentation, recruiting immune suppressor cells and expressing inhibitory molecules, thereby hindering the immune reaction through the blockade of co-stimulatory signals and activation of the immune checkpoint pathway (e.g., anti-PD-1 and its ligand PD-L1), leading to T cell anergy and exhaustion [22]. The use of ICI has significantly improved OS for patients with gastroesophageal carcinoma in advanced stages [23]. These findings suggest that neoadjuvant PD-1 blockers may elicit a potent systemic immune response, potentially eradicating residual micrometastases post-surgical removal of the primary tumour. Additionally, traditional CT has shown the ability to enhance tumour antigenicity, disrupt suppressive immune pathways and enhance effector T cell responses [24].

The mismatch repair deficient (dMMR)/microsatellite instability-high (MSI-H) phenotype, is present in about 5%–22% of gastric and GEJ ACs. Generally depended on the geographical differences, the different tumour stages and the approaches utilised to analyze the MSI status, rising to 48% in patients over 85 years old [25]. MSI-H has emerged as a significant predictive biomarker for ICIs [26].

Several clinical trials have investigated the efficacy and safety of IO in the neoadjuvant setting for resectable gastroesophageal cancer, exploring various scenarios and combinations.

### IO plus CT

The advent of IO in the treatment landscape for gastroesophageal cancer represents a significant paradigm shift, offering promising therapeutic perspectives. Initial trials combining IO with CT have shown considerable potential. One pioneering trial in this area, PALACE-1, a phase Ib trial, enrolled 22 Chinese patients with resectable esophageal squamous cell carcinoma, regardless of PD-L1 status, who received preoperative Pembrolizumab alongside concurrent chemoradiotherapy. Results from this trial indicated that the combination did not prolong the timing of surgery and induced a pCR in 55.6% of resected tumours [27].

Subsequent numerous trials (Table 1) have further explored the efficacy of this combination, primarily within Asian populations. The preliminary findings of the DANTE/IKF-s633 trial, involving 295 patients with resectable gastroesophageal AC, randomly assigned patients to receive perioperative FLOT with or without atezolizumab. Within this German study, where 8% of patients exhibited MSI-H, the addition of atezolizumab led to a higher pCR rate (ypT0N0 24% versus 15%, *p* = 0.032). This discrepancy was more pronounced in the PD-L1 CPS ≥10 and MSI-H subpopulation. Importantly, differences persisted even upon excluding patients who were dMMR [28].

Conversely, the MATTERHORN Phase III trial, assessed the PD-1 inhibitor Durvalumab alongside FLOT in 948 patients with locally advanced, resectable gastric or GEJ AC. This global study includes patients from Europe 53%, Asia 19%, South America 19% and North America 9%. Durvalumab plus FLOT exhibited a statistically significant 19% in pCR compared to a 7% in the FLOT-only arm, representing a 12% absolute improvement (OR = 3.08, p < 0.00001). The OS results, which were its primary end point, has not been presented yet. Even though patients with PD-L1 expression <1% did not derive benefit from the addition of durvalumab, notable benefits were observed in both MSI-H and non-MSI-H subgroups [29]. Another Phase III trial, KEYNOTE-585, assessed perioperative CT (cisplatin-5FU or FLOT) ± pembrolizumab in locally advanced, resectable gastroesophageal AC. While pembrolizumab led to an enhanced pCR rate (12.9% versus 2.0%; p < 0·00001), this did not translate into significant improvements in either event-free and OS [30].

The ongoing phase II/III EA2174 trial, studies the benefit of adding perioperative nivolumab and ipilimumab to CT (carboplatin + paclitaxel) and RT in patients with locoregional esophageal and GEJ AC [31]. While the phase II IMAGINE trial is studying FLOT ± nivolumab ± relatlimab (an anti-LAG3 monoclonal antibody) [32]. These and other ongoing trials continue to explore novel treatment approaches in this challenging disease landscape.

### IO alone

The French single-arm multicenter phase II study, NEONIPIGA, evaluated preoperative nivolumab and ipilimumab followed by postoperative nivolumab in resectable dMMR/MSI-H gastric/GEJ AC. Among the 32 included patients, 27 (84%) completed the planned six cycles of neoadjuvant therapy. Of the 29 patients that underwent surgery, 17 (58.6%; 90% CI, 41.8 to 74.1) achieved pCR [33]. Another trial, INFINITY, a single-arm multi-cohort phase II trial, investigated the activity and safety of tremelimumab + durvalumab as neoadjuvant (Cohort 1) or definitive (Cohort 2) treatment for MSI-H, dMMR and Epstein-Barr Virus-negative resectable gastric/GEJ AC. Among the 18 patients included in Cohort 1, where patients received a 12-week treatment with tremelimumab and durvalumab followed by surgery, a pCR rate of 60% and a major-complete pathological response of 80% were observed, with PD-L1 CPS showing no association with outcomes and tumour mutation burden (TMB) demonstrating a non-significant trend of correlation with pCR [34].

Subsequently, in a multicenter single-arm phase I trial by Hasegawa et al [35] 31 patients with resectable GC underwent neoadjuvant nivolumab monotherapy, irrespective of their PD-L1 expression, MMR status or TMB. This trial showcased a major pathological response (MPR) of 16%, mostly in patients with positive PD-L1 expression, MSI-H and/or high TMB [35]. Furthermore, a single-arm prospective phase 1b trial (NATION-1907) investigated the safety profile and preliminary therapeutic efficacy of neoadjuvant PD-L1 blockade with Adebrelimab in resectable esophageal SCC. Of the 25 eligible patients, 16% had CPS >10. A MPR, was seen in 24% of the patients. Differences between responders and no responders were not associated with TMB nor MSI [36].

An ongoing 4-cohort phase II trial, IMHOTEP, will recruit endometrial, colorectal, gastric and other cancers with localised MSI-H/dMMR and treat them with a single dose of Pembrolizumab. This is one of the first clinical trials investigating perioperative ICI in localised MSI/dMMR in a tumour-agnostic setting [37].

### IO and anti-VEGF

Various targeted therapies have demonstrated efficacy in patients with advanced GC and GEJ AC, including anti-angiogenic agents and ICIs. Preclinical data have illustrated extensive immune modulatory effects within the tumour microenvironment induced by antiangiogenic agents, providing a rationale for investigating dual blockade of VEGF and immune checkpoints [38].

One trial in the locally advanced setting trying this combination was recently published by Lin et al [7, 13] The multicenter randomised, phase 2 trial (NCT04195828), 106 patients with gastric AC were randomly assigned to receive neoadjuvant camrelizumab and apatinib combined with nab-paclitaxel plus S-1 (CA-SAP) or CT SAP alone (SAP) for 3 cycles. CA-SAP was associated with a significantly higher MPR rate (33.3%) than SAP (17.0%, p  =  0.044). The CA-SAP group also had a significantly higher objective response rate (66.0% versus 43.4%, p  =  0.017) and R0 resection rate (94.1% versus 81.1%, p  =  0.042) than the SAP group. A trend toward a higher MPR rate in patients with MSI-H [7].

DRAGON IV trial is an open label, phase III trial, that studies perioperative camrelizumab combined with rivoceranib and S1 plus Oxaliplatin (SOX) versus standard of care for locally advanced resectable gastric or GEJ AC. With 360 patients randomised, the trial reported a pCR rate of 18.3% (95% CI 13.0–24.8) for SOX combined with IO and anti-VEGF therapy, compared to 5.0% (95% CI 2.3–9.3) for SOX alone, representing a statistically significant improvement of 13.7% (95% CI 7.2–20.1, p < 0.0001). Moreover, the MPR rate was 51.1% versus 37.8%, respectively [39].

Although not a randomised trial, Wang et al [40] reported in 2023 the findings of a prospective cohort study involving 73 patients with locally advanced GC. Patients were treated with PD-1 inhibitors (sintilimab, camrelizumab or toripalimab) in combination with apatinib and CT (SOX or CAPOX) or with apatinib and CT alone. The triple combination group was designated as PAC (n = 39), while the other group was labeled as AC (*n* = 34). The PAC group demonstrated a higher objective response rate compared to the AC group (74.4% versus 58.8%, *p* = 0.159). Furthermore, the PAC group exhibited a trend toward a more favourable response profile than the AC group (*p* = 0.081). Notably, progression-free survival (*p* = 0.019) and OS (*p* = 0.049) were extended in the PAC group, while DFS tended to be longer although not statistically significant (*p* = 0.056) [40].

## Discussion

The management of gastroesophageal cancers presents significant challenges, necessitating a multidisciplinary approach that often involves CT, radiation and surgery [41, 42]. In the locally advanced setting, neoadjuvant therapy offers several advantages, including the opportunity to assess tumour response and tailor subsequent treatments accordingly. It also holds the potential to improve R0 resection rates and enhance compliance with systemic therapy, while providing valuable insights into tumour biology [43].

While SCC of the esophagus may benefit more from neoadjuvant radiation compared to AC [8], the treatment paradigm for gastric AC revolves around perioperative CT, as demonstrated by landmark trials like MAGIC [44] and FLOT4 [9]. The integration of neoadjuvant and perioperative therapies has shown significant progress in various combinations for esophageal, GEJ and GC eligible for resection. IO and targeted therapy represent promising avenues in this context, with ongoing research focusing on targets such as VEGFR, HER2 and PD-L1 [10].

While IO has revolutionised the treatment landscape in advanced-stage disease [23, 45], its role in neoadjuvant settings remains less defined. In locally advanced resectable esophageal SCC, incorporating IO into standard neoadjuvant chemoradiotherapy has not substantially increased the pCR but has raised concerns about increased toxicity [10]. Evidence for neoadjuvant IO is primarily derived from small-scale single-arm phase I/II trials and is not yet ready for widespread application. To notice, the precedent of a very similar tumour model, such as squamous cell carcinoma of the head and neck, from five negative phases three trials when IO was attempted to be added to chemoradiation.

In contrast, evidence for neoadjuvant IO in locally advanced gastroesophageal AC is emerging from phase III trials such as KN585 and MATTERHON. These trials have shown promising increases in pCR rates, with a combined total benefit in pCR of 10.9% and 12%, respectively [29, 30]. However, data on long-term survival outcomes remain limited. This, especially the results seen in KEYNOTE-585 questions whether pCR is an adequate subrrogate for EFS and OS.

Several challenges must be addressed before neoadjuvant IO can be widely adopted. These include identifying predictive biomarkers to guide patient selection and understanding the role of adjuvant therapy post-surgery. For instance, in the INFINITY trial, the finding that all patients who achieved pCR had negative circulating tumour DNA status before surgery [34] raises the hypothesis about the potential utility of adjuvant therapy or the necessity of surgery in this scenario. Further research is needed to confirm this hypothesis and determine the optimal course of action.

Not only IO is a field of research in the neoadjuvant setting. Targeted therapies such as trastuzumab [46] and T-Dx have shown efficacy in advanced GC [47], albeit in a limited subset of patients, only 20% of patients with GC are suitable for this targeted therapy. While trials like INNOVATION and PETRARCA reported increased MpRR of 13.7% and 13%, respectively [20, 48], with anti-HER2 treatment, further research is needed to refine treatment strategies and expand the patient population eligible for targeted therapy.

Anti-angiogenic therapy currently serves as an additional therapeutic approach for patients with advanced GC in the second-line setting, as evidenced by the RAINBOW trial, which demonstrates its ability to prolong patient survival and improve quality of life [49]%. Despite its favourable outcomes and clinical utility, further prospective randomised studies are necessary to evaluate the efficacy of drugs used in this therapy during the early stages. However, promising improvements in clinical outcomes have been observed in patients treated with CT and anti-angiogenic treatment, as reported by Lin et al [13] and Zheng [12].

While significant strides have been made in neoadjuvant therapy for gastroesophageal cancers, numerous challenges and unanswered questions remain. Even though, there is always a risk that multitargeted therapy might constitute a too expensive approach depending on the possibilities of particular medicinal facilities, further research is imperative to optimise treatment strategies, identify predictive biomarkers and expand the scope of targeted therapies to benefit a broader patient population.

## Conclusion

In conclusion, the integration of targeted therapy into neoadjuvant treatment has emerged as a focal point in the realm of gastroesophageal cancer therapy. Despite several studies yielding unsatisfactory outcomes, this treatment approach remains promising, offering the potential to evolve into a novel therapeutic regimen for gastroesophageal cancer.

## Future directions

Despite the strides made in the multimodal treatment of GC, recurrences remain prevalent. Consequently, research has shifted focus toward unraveling the onco-molecular biology mechanisms and identifying various target genes associated with pathogenesis and progression. The exploration of drugs targeting these genes has rapidly evolved within the realm of GC therapy.

While emerging targets like Claudin 18.2 are gaining traction in the advanced setting, investigations into new combinations and treatments targeting these novel markers are upcoming in the early-stage direction.

## Conflicts of interest

The authors declare no conflicts of interest.

## Funding

No funding was received for this work.

## Author contributions

Study conception and design, interpretation of results and draft manuscript preparation: B. Freile, F. Esteso.

All authors reviewed the results and approved the final version of the manuscript.

## Figures and Tables

**Table 1.** Clinical trials of IO + CT +/- RT.

Trial	Author and year	IO	Combination therapy	Control arm	Perioperative treatment	Histology	Population	*N*	Main result
MATTERHON	Janjigian, 2024	Durvalumab	FLOT	FLOT	Yes	AC	WW	948	pCR: 19% versus 7% (p < 0.00001). Asia: 19% versus 6%. Non-Asia: 19% versus 8%
KEYNOTE 58516/5/25 10:01:0016/5/25 10:01:00	Shitara, 2023	Pembrolizumab	CDDP+C+5Fu	CT	Yes	AC	WW	804	pCR: 12.9% versus 2.0% (p < 0.00001) mEFS: NR versus 25.3 m (*p* = 0.0198)
DANTE/IKF-S633	Lorenzen, 2024	Atezolizumab	FLOT	FLOT	Yes	AC	European	295	pCR: 24% versus 15% (*p* = 0.032)
PANDA	Verschoor, 2024	Atezolizumab	DOC	No	Yes	AC	Netherlands	21	pCR: 70% (CI 95%, 46%–88%)
PERFECT	Ende, 2021	Atezolizumab	CDBCA + Paclitaxel + RT	No	No	AC	Netherlands	40	pCR: 30%
NCT02918162	Rufi, 2022	Pembrolizumab	CAPOX	No	Yes	AC	American	36	pCR: 20.6%
KEYSTONE 001	Jiang, 2023	Pembrolizumab	CDDP + Paclitaxel	No	No	SCC	Asian	49	pCR: 42.2%. ORR: 95.6%
NCT03488667	Sun, 2022	Pembrolizumab	mFOLFOX	No	Yes	AC	American	35	pCR: 19%
NCT05602935	Zhong, 2024	Camrelizumab	SOX	No	Yes	AC	Asian	29	pCR: 10.3% (3/29)
BRES-1	Yang, 2023	Camrelizumab	CDDP + Nab-Pacltaxel	No	No	SCC	Asian	19	pCR: 45%
ChiCTR2000030610	Liu et al [22], 2022	Camrelizumab	FLOT	FLOT	No	AC	Asian	61	pCR: 11.5% versus 4.8%. R0: 100% versus 90.5%
NICE	Liu et al [22], 2022	Camrelizumab	CBDCA + Nab-Pacltaxel	No	No	SCC	Asian	60	pCR: 39.2%
NIC-ESCC2019	Liu et al [22], 2022	Camrelizumab	CDDP + Nab-Pacltaxel	No	No	SCC	Asian	56	pCR: 35.3% (95% CI, 21.7%–48.9%)
Neo-PLANET	Tang, 2022	Camrelizumab	CAPOX + RT	No	No	AC	Asian	36	pCR: 33.3% (95% CI, 18.6–51.0)
NCT04460066	Li et al [39], 2023	Socazolizumab	CDDP + Nab-Paclitaxel	CT	No	SCC	Asian	64	pCR: 41.4% versus 27.6% (*p* = 0.311)
NCT04890392	Yin, 2022	Tislelizumab	SOX	No	No	AC	Asian	32	pCR: 25%
CRISEC	Yang, 2022	Tislelizumab	CDDP + Nab-Paclitaxel + RT	No	No	SCC	Asian	30	pCR: 46.7%
TD-NICE	Yan, 2022	Tislelizumab	CBDCA + Nab-Paclitaxel	No	No	SCC	Asian	45	pCR: 50%
NCT04065282	Jiang, 2022	Sintilimab	CAPOX	No	No	AC	Asian	36	pCR: 19.4%
SIN-ICE	Duan, 2021	Sintilimab	CT (platinum based)	No	No	SCC	Asian	23	pCR: 35.5%
NCT04341857	Li et al [27], 2021	Sintilimab	FLOT	No	Yes	AC	Asian	20	pCR: 18.8%
NCT03288350	Alcindor, 2020	Avelumab	mDCF	No	Yes	AC	Canadian	28	pCR: 22%
NCT03490292	Uboha, 2022	Avelumab	CBDCA + Paclitaxel + RT	Yes	No	AC + SCC	American	22	pCR: 26%
NCT02844075	Lee, 2019	Pembrolizumab	CBDCA + Paclitaxel + RT	No	Yes	SCC	Asian	28	pCR: 46.1%, 1 2m-OS 80.8%, 18m-OS 73.1%
PALACE-1	Li et al [27], 2021	Pembrolizumab	CBDCA + Paclitaxel + RT	No	No	SCC	Asian	20	pCR: 55.6%
PEN-ICE	Duan, 2022	Pembrolizumab	CT (platinum based)	No	No	SCC	Asian	18	pCR: 46.2%
ESONICT-2	Gao, 2022	Toripalimab	CDDP + Docetaxel	No	No	SCC	Asian	20	pCR: 16.7%
SCALE-1	Jiang, 2022	Toripalimab	CBDCA + Paclitaxel + RT	No	No	SCC	Asian	22	pCR: 55%
NCT04437212	Xu, 2022	Toripalimab	CDDP + Paclitaxel + RT	No	Yes	SCC	Asian	20	pCR: 54%
NCT03165994	Ko, 2022	Sotigalimab	CBDCA + Paclitaxel + RT	No	No	AC + SCC	Asian	34	pCR 36%

CDDP: cisplatin; C: capecitabine; 5Fu: 5Fluorouracilo; CT: chemotherapy; WW: world wide; pCR: pathological complete response; mEFS: median event free survival; NR: not reached; m: months; FLOT: 5fluorouracilo + leucovorin + oxaliplatin + docetaxel; R0: complete resection; SCC: squamous cell carcinoma; CAPOX: capecitabine + oxaliplatin; RT: radiotherapy; CI: confident interval; SOX: S1 + oxaliplatin; mDCF: cisplatin + docetaxel + 5fluorouracil; FOLFOX: 5fluorouracilo + oxaliplatin; IO: immunotherapy; CBDCA: carboplatin; mOS: median overall survival
